# Myricetin mitigates motor disturbance and decreases neuronal ferroptosis in a rat model of Parkinson’s disease

**DOI:** 10.1038/s41598-024-62910-6

**Published:** 2024-07-02

**Authors:** Si-Chun Gu, Zhi-Guo Xie, Min-Jue Gu, Chang-De Wang, Li-Min Xu, Chen Gao, Xiao-Lei Yuan, You Wu, Yu-Qing Hu, Yang Cao, Qing Ye

**Affiliations:** 1 Department of Neurology, Longhua Hospital, Shanghai University of Traditional Chinese Medicine, 725 South Wanping Road, Shanghai, 200032 China; 2 Department of Gynecology, Yueyang Hospital of Integrated Traditional Chinese and Western Medicine, Shanghai University of Traditional Chinese Medicine, Shanghai, 200437 China; 3 Shanghai TCM-Integrated Hospital, Shanghai University of Traditional Chinese Medicine, 230 Baoding Road, Shanghai, 200082 China

**Keywords:** Parkinson’s disease, Myricetin, Ferroptosis, Nrf2, Gpx4, Biochemistry, Cell biology, Molecular biology

## Abstract

Ferroptosis is an iron-dependent cell death form characterized by reactive oxygen species (ROS) overgeneration and lipid peroxidation. Myricetin, a flavonoid that exists in numerous plants, exhibits potent antioxidant capacity. Given that iron accumulation and ROS-provoked dopaminergic neuron death are the two main pathological hallmarks of Parkinson’s disease (PD), we aimed to investigate whether myricetin decreases neuronal death through suppressing ferroptosis. The PD models were established by intraperitoneally injecting 1-methyl-4-phenyl-1,2,3,6-tetrahydropyridine (MPTP) into rats and by treating SH-SY5Y cells with 1-methyl-4-phenylpyridinium (MPP^+^), respectively. Ferroptosis was identified by assessing the levels of Fe^2+^, ROS, malondialdehyde (MDA), and glutathione (GSH). The results demonstrated that myricetin treatment effectively mitigated MPTP-triggered motor impairment, dopamine neuronal death, and α-synuclein (α-Syn) accumulation in PD models. Myricetin also alleviated MPTP-induced ferroptosis, as evidenced by decreased levels of Fe^2+^, ROS, and MDA and increased levels of GSH in the substantia nigra (SN) and serum in PD models. All these changes were reversed by erastin, a ferroptosis activator. In vitro, myricetin treatment restored SH-SY5Y cell viability and alleviated MPP^+^-induced SH-SY5Y cell ferroptosis. Mechanistically, myricetin accelerated nuclear translocation of nuclear factor E2-related factor 2 (Nrf2) and subsequent glutathione peroxidase 4 (Gpx4) expression in MPP^+^-treated SH-SY5Y cells, two critical inhibitors of ferroptosis. Collectively, these data demonstrate that myricetin may be a potential agent for decreasing dopaminergic neuron death by inhibiting ferroptosis in PD.

## Introduction

Parkinson’s disease (PD) is one of the most frequent neurodegenerative disorders worldwide that currently has no cure^1,2^ and affects about one percent of the population over 60 years and five percent of the population over 85 years^3^. PD is characterized by the massive loss of dopaminergic neurons in the substantia nigra pars compacta (SNc) and iron deposition in the basal ganglia and SN^4,5^. Nowadays, medicines help alleviate the symptoms of PD patients. The most common medication is Levodopa, which enhances dopamine levels in the brain, thereby improving symptoms^6,7^. However, there is currently no reliable treatment to hamper the progression of PD.

For several decades, it has been observed that iron deposits in various brain areas in PD, particularly in the basal ganglia and SN^8^. Excessive cellular iron mediates the production of neurotoxic metabolites of dopamine^9^ and promotes reactive oxygen species (ROS) formation, which exacerbates mitochondrial dysfunction^10^, mediates covalent modifications to DNA^11^, and directly induces protein oxidative modification^12^. The iron overload and resultant increased ROS production ultimately lead to cellular senescence or iron-triggered cell death^13^. Ferroptosis has been established as a novel cell death form characterized by Fe^2+^ overload, ROS overproduction, and lipid peroxidation^14^. Of note, ferroptosis acts as a contributor to the progressive degeneration of dopaminergic neurons in PD^15^. These observations represent strong support for the roles of ferroptosis in dopaminergic cell survival. Therefore, it might be an effective approach for PD treatment by regulating ferroptosis-dependent degeneration of dopaminergic neurons.

In recent years, medicinal plant-derived natural products possessing antioxidant potential have been recommended as hopeful options for different kinds of diseases. Compounds with unique neuroprotective effects, such as rosmarinic acid and carnosic acid, have shown pharmacological use in several neurodegenerative disorders by decreasing neuronal cell damage^16–18^. Chicoric acid, an important polyphenolic acid compound found in plants like chicory, lettuce, and dandelion, has shown positive effects on preventing neurodegenerative progression^19^. Moreover, curcumin has been shown to ameliorate motor deficits and provide protection against dopaminergic neuron loss^20^.

Myricetin, a naturally extracted flavonoid, was noted for its antioxidant, anti-inflammatory, and anticancer abilities^21,22^. According to previous studies, myricetin has the ability to hamper the progression of PD through mechanisms associated with anti-oxidation and inhibition of iron accumulation-associated pathologies^23^. A study by Molina-Jiménez et al. found that myricetin incubation acts against rotenone-induced cell loss in SH-SY5Y cells through decreasing hydrogen peroxide and superoxide anion production^24^. Additionally, the in vivo study demonstrated that myricetin treatment significantly suppresses 6-hydroxydopamine-induced iron-staining cell proliferation in the SN^25^. However, the regulatory roles of myricetin in neuronal ferroptosis remain unclear. Based on these findings, we aimed to investigate whether myricetin decreases neuronal death by inhibiting ferroptosis.

## Materials and methods

### Reagents

SH-SY5Y cells were obtained from the procell (Wuhan, China). MPTP hydrochloride (Cat# ST1020), PFA (Cat# P0099), and RIPA lysis buffer (Cat# P0013B) were obtained from Beyotime (Shanghai, China). Myricetin (Cat# SM8390) and hematoxylin (Cat# G1080) were obtained from Solarbio (Beijing, China). Primary antibodies against α-Syn (Cat# ab212184), Nrf2 (Cat# ab137550), Gpx4 (Cat# ab125066), β-actin (Cat# ab8227), Lamin B1 (Cat# ab16048), and tyrosine hydroxylase (Cat# ab137869), secondary antibody goat anti-rabbit IgG H&L (Cat# ab6721), and DCFDA (Cat# ab113851) were obtained from Abcam (Cambrige, MA, USA). Erastin (Cat# HY-15763) and Ferrostatin-1 (Fer-1, Cat# HY-100579) were obtained from MCE (NJ, USA). DMEM (Cat# 11960044), FBS (Cat# 16140071), and penicillin–streptomycin (Cat# 15140148) were obtained from Thermo Fisher (MA, USA). Xylene (Cat# X112050), Tween-20 (Cat# T104863), and ethanol (Cat# E111993) were obtained from Aladdin (Shanghai, China). Lipid peroxidation MDA assay kit (Cat# S0131S, Beyotime), total glutathione assay kit (Cat# S0052, Beyotime), Pierce BCA protein assay kit (Cat# A55864, Thermo Fisher), HRP DAB kit (Cat# 34,002, Thermo Fisher), and iron assay kit (Cat# ab83366, Abcam) were used in the study.

### A rat model of PD

The animal studies were approved by the Animal Ethics Committee of Longhua Hospital, Shanghai University of Traditional Chinese Medicine (No. 2018–0036) in accordance with the ARRIVE guidelines to minimize animal suffering. Eight week-old male SD rats (230–250 g) were obtained from Charles River (Shanghai, China) and maintained in pathogen-free facilities at conditions of 20–24 °C and 40–60% relative humidity with a 12 h light/dark cycle. Rats were reared ad-libitum with water and standard food. To establish the PD model, rats were intraperitoneally injected with MPTP once daily at a dose of 30 mg/kg/day for five consecutive days. Control rats were injected with the vehicle without MPTP.

Myricetin was intragastrically administered at 25 mg/kg once a day from day 1 to day 14, according to previous studies^26^ and our preliminary experiment. Erastin (15 mg/kg) was delivered intranasally by pipette to rats immediately after MPTP injection, as previously described^27^. All rats were weighed once a week from day 7. Rats were euthanized using a pentobarbital overdose followed by cervical dislocation after 4 weekends, and the SN were harvested for the following analysis.

### Rotarod test

A rotarod cylinder was set to gradually accelerate from 4 to 40 rotations per minute (rpm) over 5 min. Rats were pre-trained to learn how to maintain their position on the rotarod. The time the rats stayed on the rotarod until they dropped or grasped the device for more than two rotations without continuing to walk was measured. Three rotarod measurements per test were made.

### Balance beam test

A square cross-section wooden stick (1 m in length and 2.5 cm in width) was positioned horizontally 60 cm above the ground. Prior training was provided to rats for balance beam locomotion. Until they fall or hold for a minute, score the rats according to the following scoring criteria: Able to jump and walk without falling scores 6; A score of 5 is assigned when the ability to walk and jump is demonstrated with a fall probability of less than one in two; A score of 4 is assigned when the ability to walk and jump is demonstrated with a fall probability of more than one in two; Able to walk with the unaffected hind limb but be unable to move with the affected paralyzed hind limb scores 3; Able to sit but be unable to walk scores 2; and fall from the balance beam scores 1. The scoring was achieved across triplicate experiments.

### Foot fault test

A 50 cm × 40 cm mesh screen with a density of 2 cm × 2 cm was positioned 30 cm above the ground. Rats were pre-trained to walk on the mesh. In the formal test, rats were permitted to ambulate for 1 min, and the foot fault when the foot misstepped and fell through the space was recorded. The foot fault value was calculated by the ratio of foot falls to total steps multiplied by 100%. Three data points were collected for every individual rat.

### Cell culture and treatment

The human SH-SY5Y cell line was maintained in a 5% CO_2_ incubator at 37 °C in DMEM containing 10% FBS. Cells were incubated with MPP + (0.4 mM), myricetin (50 μM), erastin (1 μM) or Fer-1 (1 μM) for 24 h, and then the following analyses were carried out.

### Western blotting

Total protein was isolated from the SN tissues and SH-SY5Y cells with RIPA lysis buffer and its concentration was assessed with a Pierce BCA protein assay kit. The sample of 20 μg was separated by 10% SDS-PAGE and then transferred to PVDF membranes. After blocking with 5% non-fat milk solution for 2 h at room temperature, the membrane was incubated with the primary antibody overnight at 4 °C. Then the membrane was incubated with a secondary antibody for 1 h at room temperature for immunoblotting. The membranes were assayed by ChemiDox XRS (Bio-Rad) and the pictures were analyzed using Image J (NIH, MD, USA). β-actin and lamin B1 were used for normalization.

### Immunohistochemical analysis (IHC)

Tissue from the SN was fixed with 4% PFA at 4 °C overnight. Then the tissue was encased in paraffin and sectioned at 4 µm thickness. These sections were dewaxed in xylene, hydrated using a series of ethanol solutions, and incubated with 2% Tween-20 for 20 min. Then the slices were treated with citrate buffer (pH 6.0) for 15 min at 108 °C to repair antigen and incubated with 3% hydrogen peroxide for 15 min at room temperature. Following a wash with PBS, sections were blocked with normal goat serum and incubated with the primary antibody and the HRP-labeled secondary antibody. Finally, slices were visualized using the HRP DAB kit and re-stained with hematoxylin.

### Intracellular ROS assay

ROS levels in the SN tissue and SH-SY5Y cells were detected using DCFDA. The SN tissue was homogenized with a glass homogenizer. Then the tissue suspension was incubated with DCFDA for 30 min. The fluorescence intensity was measured using a microplate reader. SH-SY5Y cells were incubated with 20 μM DCFDA for 1 h and then fixed with 4% PFA for 10 min after washing with PBS. ROS fluorescence intensity was analyzed with a fluorescent microscope (Nikon, Tokyo, Japan) and quantified using Image J (NIH).

### *Fe*^*2*+^*levels*

Total iron contents in the SN tissues and SH-SY5Y cells were determined using an iron assay kit. The SN tissue and SH-SY5Y cells were homogenized in iron assay buffer and centrifuged (13,000 rpm, 4 °C) for 5 min. Then the supernatant was incubated with an iron reducer for 30 min and an iron probe for 60 min at 37 °C in the dark. Absorbance was gauged at 593 nm with a microplate reader (Thermo Fisher Scientific).

### Assessment of MDA and GSH

The level of MDA in SN tissue and SH-SY5Y cells was detected using a lipid peroxidation MDA assay kit. The MDA levels were measured using a microplate reader at 532 nm. The level of MDA in SN tissue and SH-SY5Y cells was detected using a total glutathione assay kit. The GSH levels were assessed with a microplate reader at 412 nm.

### Statistics

The data represent at least three independent replicates and were displayed as the mean ± SD. Intergroup comparisons were analyzed using the student’s *t* test. Multi-group comparisons were analyzed using one‐way ANOVA (Scheffé test). *p* value less than 0.05 was considered statistically significant.

### Ethics approval and consent to participate

The animal studies were approved by the Animal Ethics Committee of Longhua Hospital, Shanghai University of Traditional Chinese Medicine (No. 2018-0036) in accordance with the ARRIVE guidelines to minimize animal suffering.

### Methods statement

All methods were carried out in accordance with relevant guidelines and regulations.

## Results

### Myricetin mitigated MPTP-induced motor impairment

**Figure 1:**
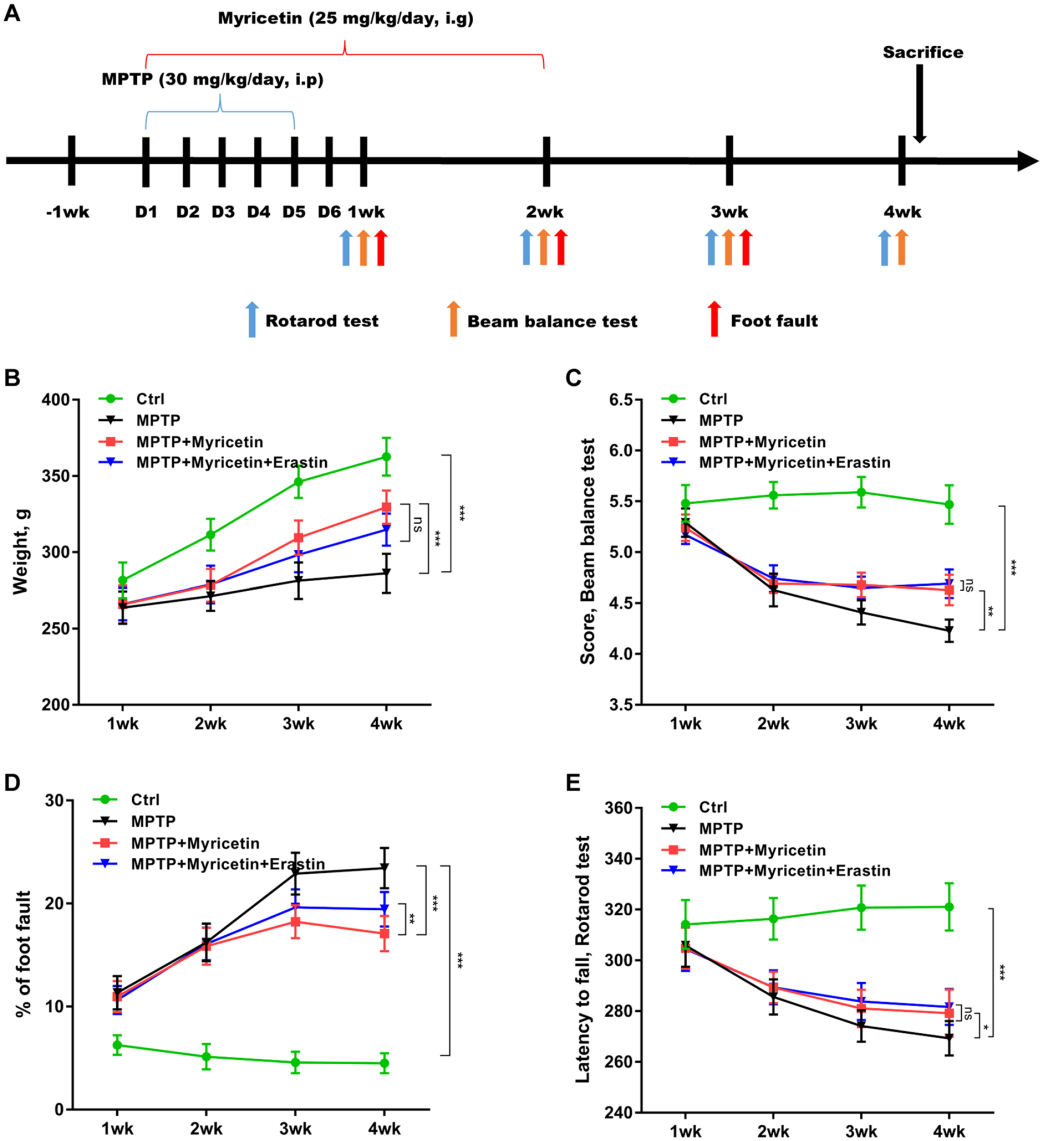
Myricetin mitigated MPTP-induced motor impairment. (**A**) The outline of the experimental procedure. (**B**) The weight gain of different groups of rats was surveyed at weeks 1, 2, 3, and 4. (**C**) The effects of myricetin on ameliorating the behavioral impairment caused by MPTP were assessed using the beam balance test. (**D**) The effects of myricetin on decreasing the foot fault numbers caused by MPTP were assessed using the foot fault test. (**E**) The effects of myricetin on alleviated the lessened latency to fall caused by MPTP were assessed using the rotarod test. n = 10, ***p < 0.001, **p < 0.01, *p < 0.05, ns, no significant.

To explore the biological effects of myricetin on improving motor impairment in PD, a rat model of PD was established by intraperitoneally injecting MPTP, and then intragastrically administered with myricetin (Fig. 1A). Figure 1B revealed that body weight gain was inhibited by MPTP, whereas myricetin treatment effectively restored body weight gain in PD rats. In the beam balance test, myricetin treatment significantly ameliorated the behavioral impairment caused by MPTP (Fig. 1C). In the foot fault test, MPTP resulted in a dramatic increase in foot fault numbers, whereas the change was blocked by myricetin (Fig. 1D). In the rotarod test, myricetin effectively alleviated the lessened latency to fall caused by MPTP (Fig. 1E). Significantly, erastin treatment damaged the effects of myricetin on decreasing foot fault counts in the foot fault test (Fig. 1D). In the beam balance test and rotarod test, no significant differences were observed after erastin treatment compared with myricetin alone in the MPTP-treated rats (Fig. 1C and E).

### Myricetin decreased MPTP-induced dopamine neuronal death and α-Syn accumulation

**Figure 2:**
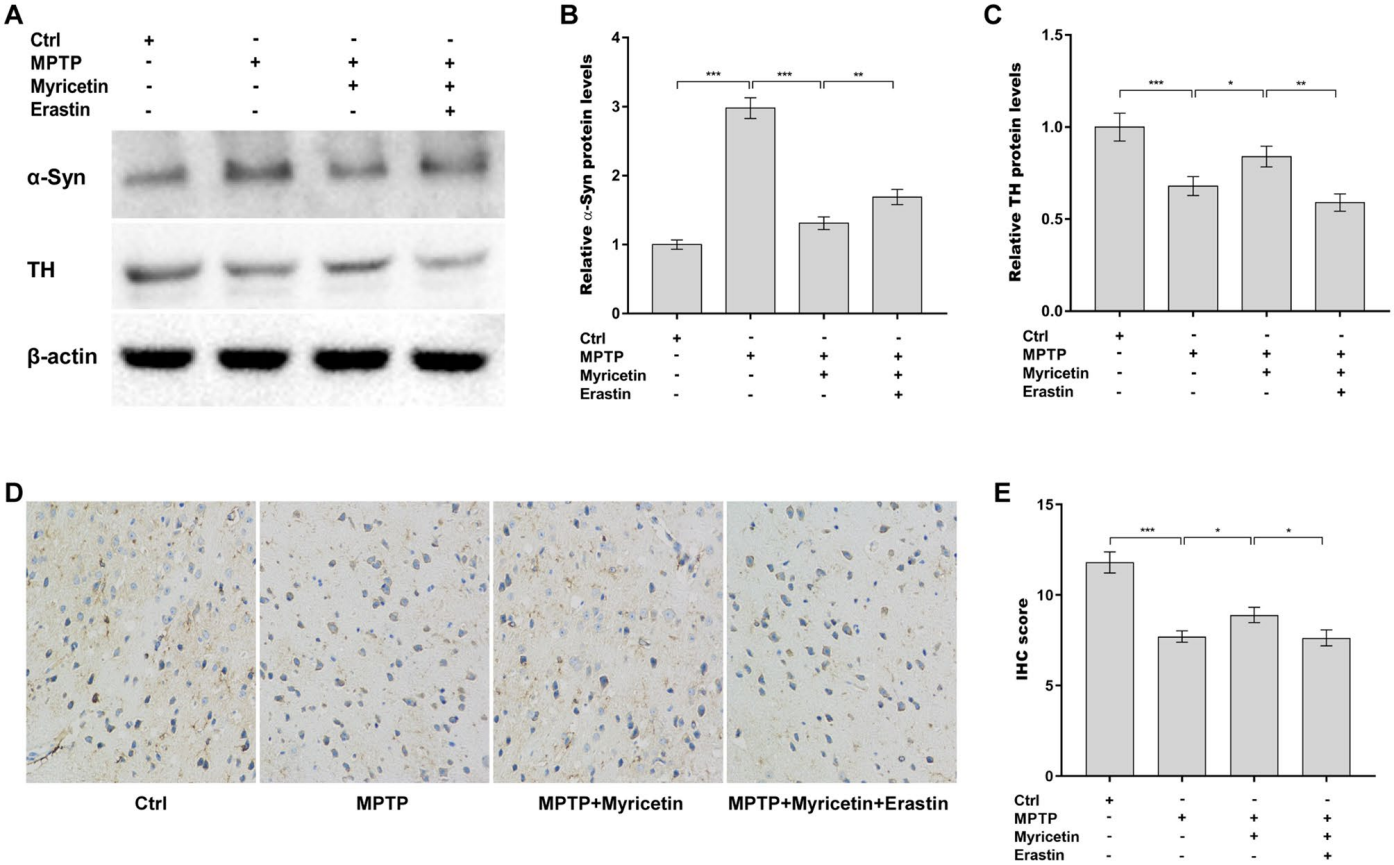
Myricetin decreased MPTP-induced dopamine neuronal death and α-Syn accumulation. (**A**) The effects of MPTP, myricetin, and erastin on the regulation of α-Syn and TH protein expression in the SN were assessed using western blot assay. Quantitative analysis of α-Syn (**B**) and TH (**C**) protein levels shown in (**A**). (**D** and **E**) IHC analysis of TH expression in the SN of different groups of rats. Magnification, × 200. n = 7, ***p < 0.001, **p < 0.01, *p < 0.05.

To assess the roles of myricetin in regulating dopamine neuronal loss and α-Syn accumulation in PD models, the expression of tyrosine hydroxylase (TH) and α-Syn was measured in the SN in a rat model of PD using western blotting and IHC assays. Myricetin inhibited the MPTP-induced increase in α-Syn protein levels in the SN (Fig. 2A and B). Meanwhile, TH protein expression was restored by myricetin in MPTP-treated rats (Fig. 2A and C). Furthermore, the IHC assay revealed that the TH-positive cells were increased in the SN after myricetin administration in MPTP-treated rats (Fig. 2D and E). All these effects were blocked by erastin (Fig. 2A–E). These data suggest that myricetin decreased MPTP-provoked dopamine neuronal loss and α-Syn accumulation by inhibiting ferroptosis.

### Myricetin mitigated MPTP-induced ferroptosis

**Figure 3:**
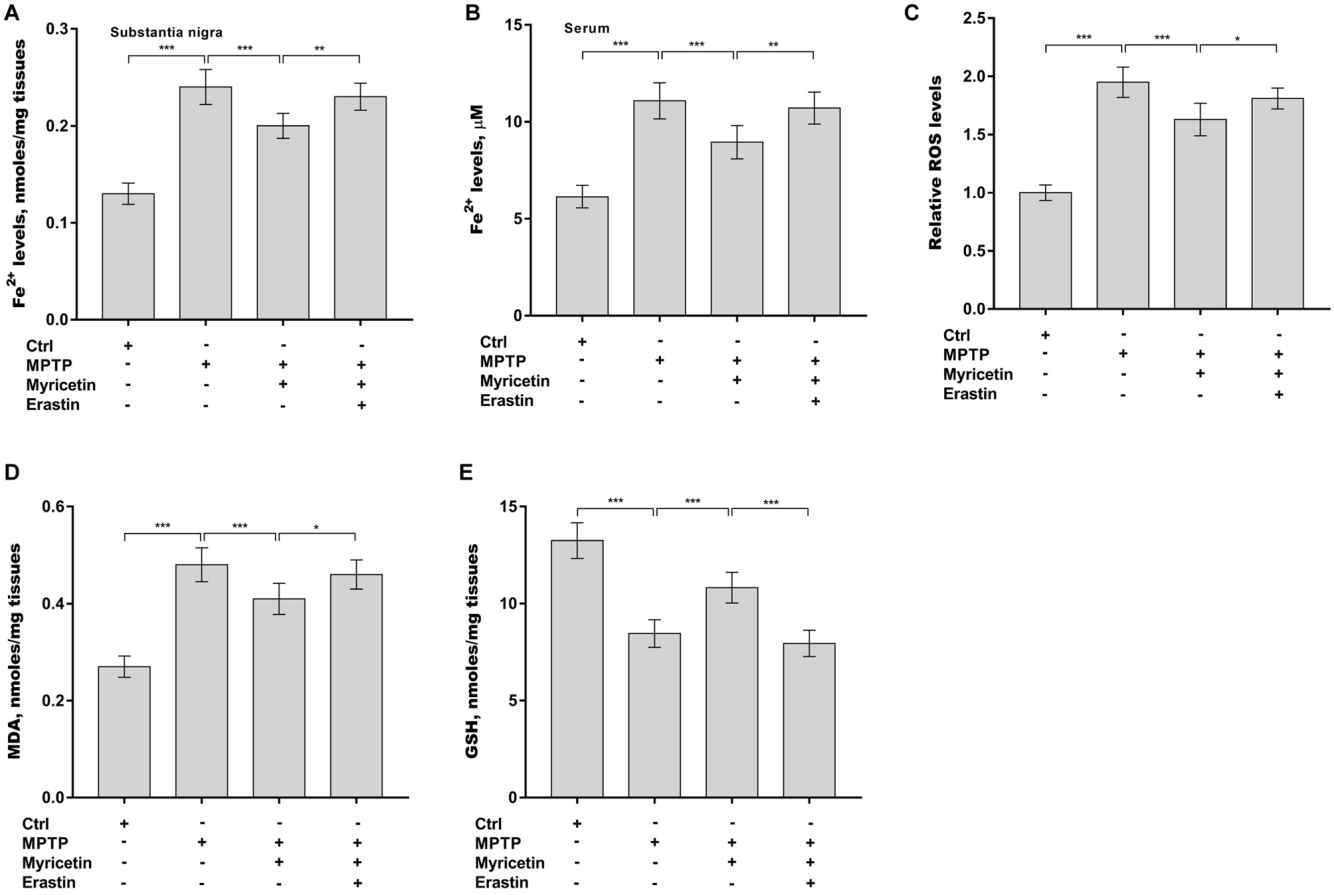
Myricetin mitigated MPTP-induced ferroptosis. (**A**) The Fe^2+^ levels in the SN (**A**) and serum (**B**) of different groups of rats were assessed by a commercial kit. The levels of ROS (**C**), MDA (**D**), and GSH (**E**) in the SN of different groups of rats were assessed by corresponding commercial kits. n = 7, ***p < 0.001, **p < 0.01, *p < 0.05.

The role of myricetin in regulating ferroptosis in PD rats was next investigated. Given that iron overload is a main pathological hallmark of PD, the Fe^2+^ levels were first assessed in the SN and serum after treatment with myricetin alone or combined with erastin in PD rats. Figure 3A and B revealed that the Fe^2+^ levels in the SN and serum were markedly increased after MPTP treatment, whereas myricetin effectively inhibited the increase. The roles of myricetin in regulating MPTP-induced oxidative stress in PD rats were further assessed. As displayed in Fig. 3C, the ROS levels were significantly elevated in the SN after treatment with MPTP, whereas these changes were reversed by myricetin. Additionally, myricetin alleviated MPTP-induced increases in MDA levels (Fig. 3D) and MPTP-caused decreases in GSH levels in the SN (Fig. 3E). All these effects were blocked by erastin (Fig. 3A–E). These data demonstrate that myricetin mitigates MPTP-induced ferroptosis.

### Latency to fall was correlated with ferroptosis

**Figure 4:**
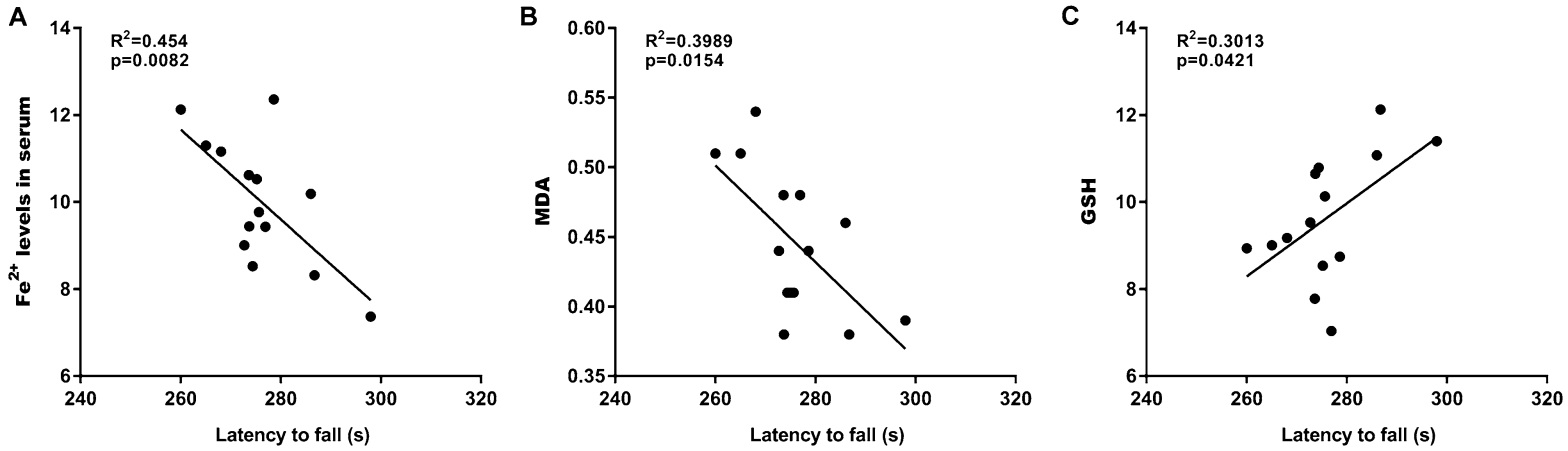
Latency to fall was correlated with ferroptosis. The correlations of latency to fall in the rat model of PD (n = 7) and myricetin-treated PD rats (n = 7) with the levels of Fe^2+^ in serum (**A**), MDA (**B**), and GSH (**C**) were analyzed.

The relationships between the latency to fall and ferroptosis markers (Fe^2+^, MDA, and GSH) were next analyzed. Although no significant association of the latency to fall with the Fe^2+^ levels was observed in the SN (data not shown), a negative correlation was found between the latency to fall and both the serum Fe^2+^ levels (Fig. 4A) and the MDA content in the SN (Fig. 4B). Additionally, the latency to fall was positively associated with the GSH content in the SN (Fig. 4C).

### Myricetin inhibited MPP^+^-induced SH-SY5Y cell ferroptosis

**Figure 5:**
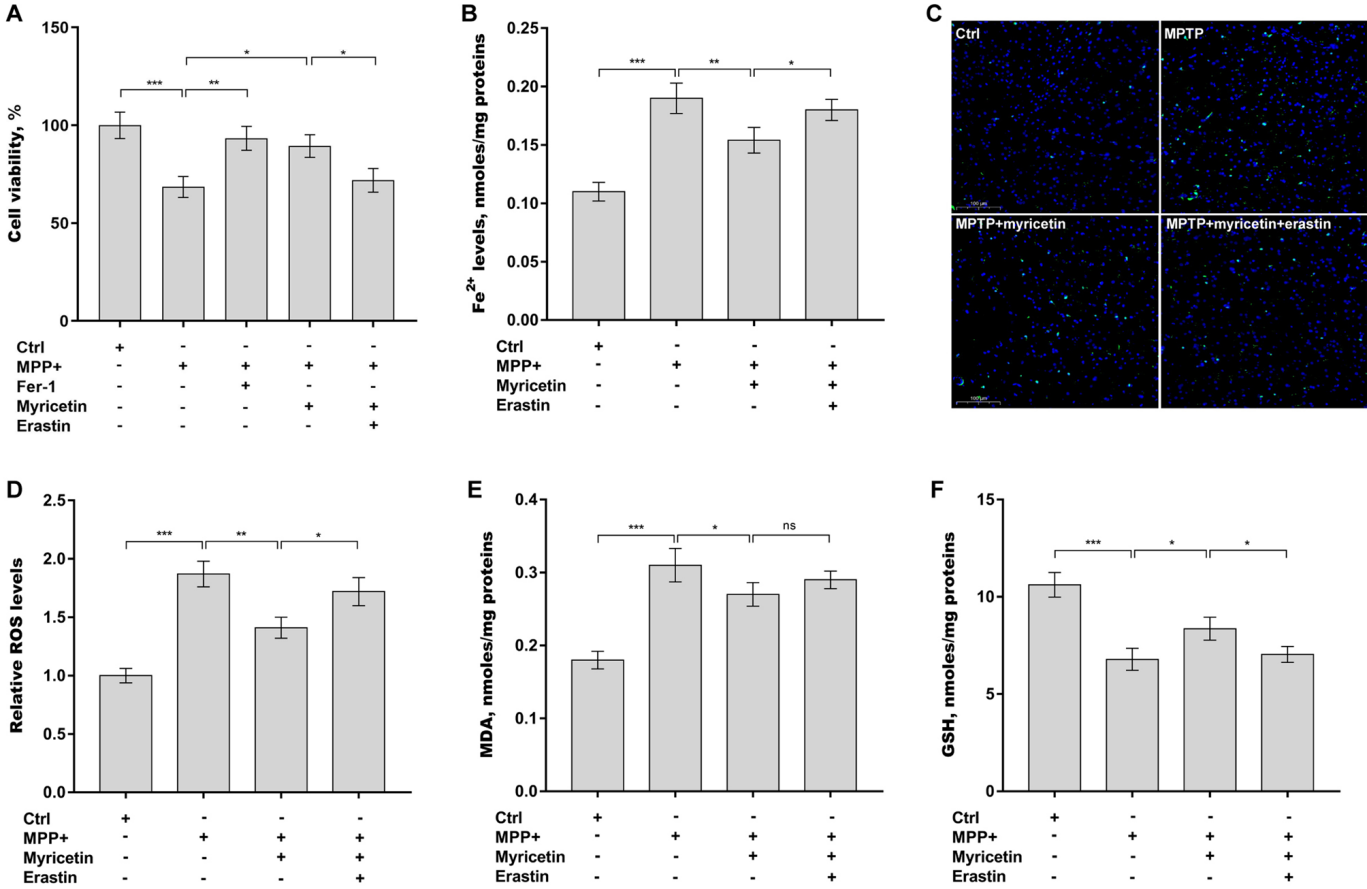
Myricetin inhibited MPP^+^-induced SH-SY5Y cell ferroptosis. SH-SY5Y cells were treated with 0.4 mM of MPP^+^ alone or combined with myricetin (50 µM), erastin (1 µM), or Fer-1 (1 µM), and then cell viability (**A**), Fe^2+^ (**B**), ROS (**C** and **D**), MDA (**E**), and GSH (**F**) levels were assessed by CCK-8 reagent and corresponding commercial kits, respectively. n = 3, ***p < 0.001, **p < 0.01, *p < 0.05, ns, no significant.

The roles of myricetin in regulating ferroptosis were next verified in SH-SY5Y cells after treatment with MPP^+^. Firstly, Fer-1 (a selective ferroptosis inhibitor) and myricetin treatment restored SH-SY5Y cell viability, which was suppressed by MPP^+^ (Fig. 5A). Secondly, myricetin decreased Fe^2+^ (Fig. 5B) and ROS (Fig. 5C and D) levels in SH-SY5Y cells, which were elevated by MPP^+^ (Fig. 5B–D). Thirdly, myricetin alleviated the MPP^+^-induced increases in MDA levels (Fig. 5E) and MPP^+^-caused decreases in GSH levels in SH-SY5Y cells (Fig. 5F). All these effects were reversed by erastin (Fig. 5A–F). These results demonstrate that myricetin alleviates MPP^+^-induced SH-SY5Y cell ferroptosis.

### Myricetin accelerated the nuclear translocation of Nrf2 and subsequent Gpx4 expression in MPP^+^-treated SH-SY5Y cells

**Figure 6:**
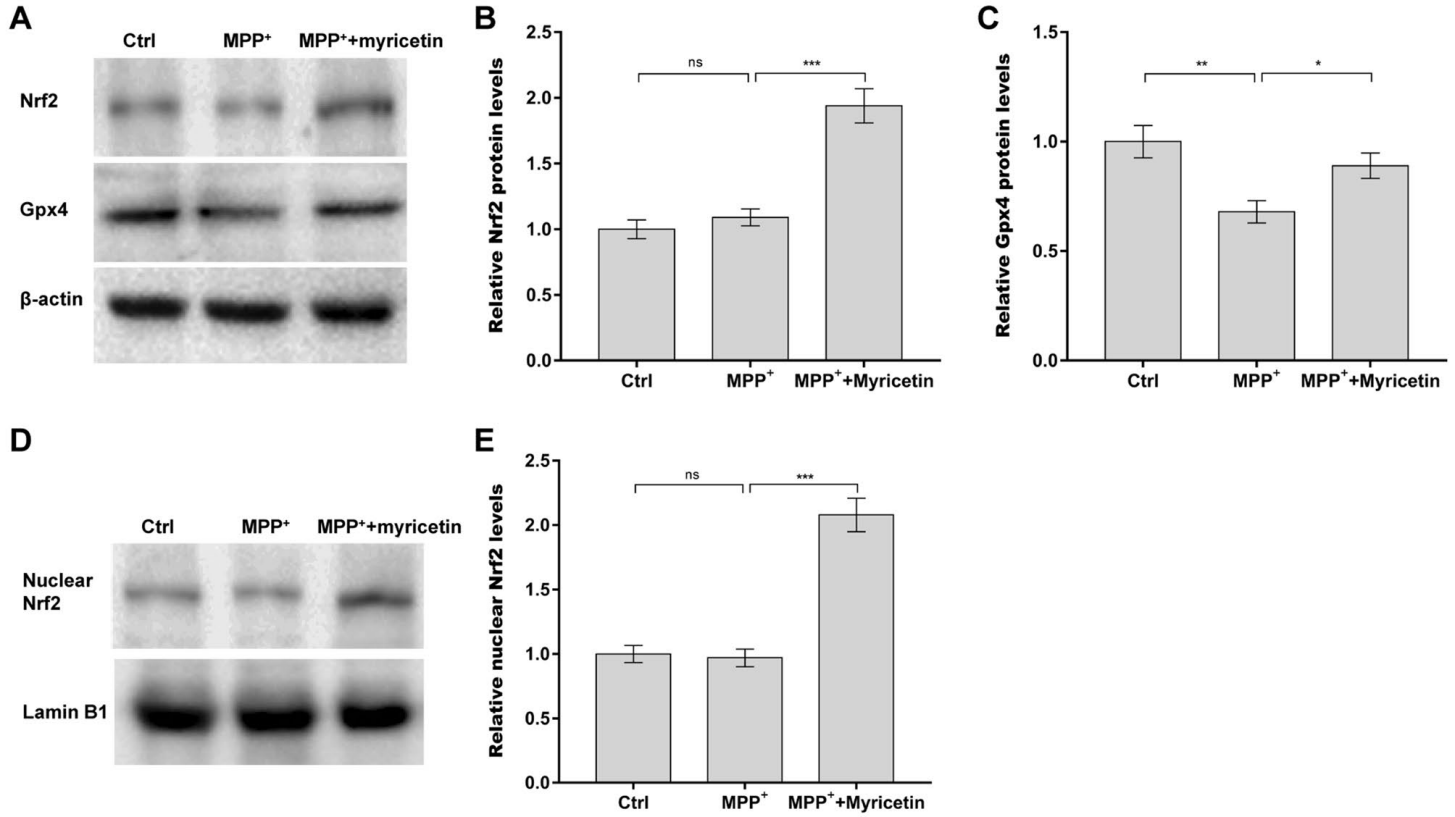
Myricetin accelerated the nuclear translocation of Nrf2 and subsequent Gpx4 expression in MPP^+^-treated SH-SY5Y cells. SH-SY5Y cells were treated with 0.4 mM of MPP^+^ alone or combined with 50 µM of myricetin, and then total Nrf2 (**A** and **B**), total Gpx4 (**A** and **C**), and nuclear Nrf2 (**D** and **E**) protein levels were assessed by western blot assay. n = 3, ***p < 0.001, **p < 0.01, *p < 0.05, ns, no significant.

The Nrf2 pathway and Gpx4 expression are two critical inhibitors of ferroptosis^28,29^. Emerging evidence has demonstrated that some natural active products in plants (DL-3-*n*-butylphthalide, quercetin, etc.) exhibit protective effects against PD in rats by regulating Nrf2/Gpx4-dependent ferroptosis^30,31^. Therefore, the roles of myricetin in activating the Nrf2 pathway and increasing Gpx4 expression were next investigated in SH-SY5Y cells after MPP^+^ treatment. To this end, SH-SY5Y cells were incubated with MPP^+^ alone or combined with myricetin, and then total and nuclear Nrf2 protein levels were measured using western blot assay. Figure 6A and B showed that there were no changes in total Nrf2 protein expression in SH-SY5Y cells after exposure to MPP^+^, whereas myricetin treatment increased total Nrf2 expression. Myricetin also markedly accelerated nuclear translocation of Nrf2 in SH-SY5Y cells in the presence of MPP^+^ (Fig. 6D and E). The protein expression of Gpx4 in SH-SY5Y cells was next measured after treatment with MPP^+^ and myricetin. Figure 6A and C showed that MPP^+^ caused a significant decrease in Gpx4 protein, whereas myricetin treatment restored Gpx4 expression in MPP^+^-treated SH-SY5Y cells.

## Discussion

PD is a neurodegenerative disorder that results in symptoms such as tremor, bradykinesia, and muscle stiffness in patients^32^. Recently, there has been a growing interest in the use of natural compounds derived from plants to alleviate the progression of PD^33^. Myricetin, a flavonoid that exists in numerous plants, displays an array of neuroprotective properties, positioning it as a potential contender for PD treatment^34^. For instance, myricetin demonstrates antioxidant properties, helping to attenuate ROS production in SH-SY5Y cells^35^. Given the role of ROS accumulation in inducing the oxidation of dopamine and the subsequent dopaminergic neuron death^36^ and the implication of neurodegeneration in the development of PD, we sought to investigate the potential regulatory role of myricetin in PD. In the current study, we demonstrated that: (1) Myricetin mitigated MPTP-induced motor impairment; (2) Myricetin decreased MPTP-induced dopamine neuronal death and α-Syn accumulation; (3) Myricetin mitigated MPTP-induced ferroptosis; (4) Latency to fall was correlated with ferroptosis; (5) Myricetin inhibited MPP^+^-induced SH-SY5Y cell ferroptosis; (6) Myricetin accelerated the nuclear translocation of Nrf2 and subsequent Gpx4 expression in MPP^+^-treated SH-SY5Y cells. These findings demonstrate that myricetin may be a promising agent for decreasing dopaminergic neuron death by inhibiting ferroptosis in PD.

ROS in the SN of PD patients are produced through several mechanisms, including impairment of the electron transport chain, exposure to environmental toxins, and genetic mutations^37–39^. Antioxidant system deficits, such as the reduction in glutathione production^40^, a decrease in catalase levels^41^, and dysfunction of superoxide dismutase 1^42^, contribute to an imbalance of ROS production and detoxification. Excessive production of ROS leads to oxidative stress, which is implicated in the death of dopaminergic neurons in PD^43,44^. Evidence of cell death is observed in numerous models of PD with a well-characterized accumulation of ROS. For instance, Todd et al. have proposed that in a yeast model of PD, ROS are considered a primary inducer of apoptosis^45^. Similarly, Zhong et al. found a significant increase in ROS content within a cellular PD model, noting a positive correlation with enhanced pyroptosis^46^. In this study, we demonstrated a correlation between the latency to fall in rats and ferroptosis-related factors. Given that the loss of dopaminergic neurons results in motor impairment^47^, it is suggested that ROS-induced ferroptosis may contribute to this loss.

Plant-derived compounds contain antioxidant substances that play a role in suppressing free radical generation and therefore reducing illness caused by oxidative stress^48^. Mounting studies have identified natural products with anti-oxidative stress properties as an effective strategy for delaying PD progression. For instance, procyanidin, kurarinone, and crocins have been used to alleviate PD progression in animal models^49–51^. Myricetin has been considered a promising natural substance for PD due to its strong anti-oxidant properties^23,52^. In the study, we demonstrated that myricetin regulated the levels of α-syn and TH protein and mediated ROS-induced ferroptosis by regulating Nrf2/Gpx4 signaling. The Nrf2 pathway and Gpx4 expression are two critical inhibitors of ferroptosis^28,29^. Emerging evidence has demonstrated that myricetin exhibits protective effects against neurodegenerative diseases by regulating the Nrf2 signaling pathway^53^. However, few investigations have been made about the potential regulatory role of myricetin in GPX4 expression and ferroptosis. As far as we are aware, our study pioneers the revelation that myricetin has a notable inhibitory effect on ferroptosis.

Additional iron accumulation is widely reported in the SN of PD patients^54^. Iron that constitutes the labile iron pool participates in generating ROS, which further result in oxidative damage and cell death^55,56^. Therefore, ferroptosis may be the leading cause of dopaminergic neuron loss. Here, we demonstrated that myricetin exhibits an anti-PD effect by suppressing ferroptosis, indicating the potential of myricetin as an anti-PD drug for patients. However, it remains unknown whether myricetin functions in other types of cell death induced by ROS in PD, such as necroptosis^57^ and pyroptosis^58^. Besides, incorporating ferroptosis inhibitor group in animal experiments helps to confirm the role of ferroptosis in PD.

## Data Availability

All relevant data supporting the conclusions of this article is included within the manuscript.
